# Delayed contrast enhancement cardiac magnetic resonance imaging in trastuzumab induced cardiomyopathy

**DOI:** 10.1186/1532-429X-10-5

**Published:** 2008-01-22

**Authors:** Nazanin Fallah-Rad, Matthew Lytwyn, Tielan Fang, Iain Kirkpatrick, Davinder S Jassal

**Affiliations:** 1 Institute of Cardiovascular Sciences, St. Boniface Research Centre, University of Manitoba, Winnipeg, Manitoba, Canada; 2 Section of Cardiology, Department of Cardiac Sciences, University of Manitoba, Winnipeg, Manitoba, Canada; 3 Department of Radiology, University of Manitoba, Winnipeg, Manitoba, Canada

## Abstract

**Background:**

Trastuzumab (Herceptin), an antagonist to the human epidermal growth factor 2 (HER2) receptor significantly decreases the rates of breast cancer recurrence and mortality by 50%. Despite therapeutic benefits, the risk of cardiotoxicity with trastuzumab ranges from 10–15% when administered sequentially following anthraycline chemotherapy. Little is known about the utility of cardiac magnetic resonance (CMR) in the assessment of trastuzumab mediated cardiomyopathy.

**Methods and results:**

Between 2005–2006 inclusive, 160 breast cancer patients were identified at a single tertiary care oncology centre. Of the total population, 10 patients (mean age 40 ± 8 years) were identified with trastuzumab induced cardiomyopathy, based on a LVEF less than 40% on serial MUGA or echocardiography. CMR was performed in all patients to determine LV volumes, systolic function and evidence of late gadolinium enhancement (LGE). At the time of diagnosis of trastuzumab induced cardiomyopathy, the mean LVEF was 29 ± 4%. Subepicardial linear LGE was present in the lateral portion of the left ventricles in all 10 patients.

**Conclusion:**

LGE-CMR is a novel way of detecting early changes in the myocardium due to trastuzumab induced cardiotoxicity.

## Introduction

Breast cancer is a major public health concern that affects 1 in 7 women in their lifetime [1]. Anthracyclines are commonly used in the setting of adjuvant therapy in the treatment of breast cancer patients. While anthracyclines significantly improve clinical morbidity and mortality, there are notable cardiotoxic side effects [2]. Recent understanding of the biology of breast cancer has lead to the introduction of a new therapeutic agent, Trastuzumab (Herceptin), an antagonist to the human epidermal growth factor 2 (HER2) receptor, which is found in 25% of breast cancer patients [3]. When added to conventional anthracycline chemotherapy, trastuzumab significantly decreases the rates of recurrence and mortality by 50% in HER-2 positive breast cancer patients [4-6]. Despite therapeutic benefits however, the risk of cardiotoxicity with trastuzumab ranges from 10–15% when administered in combination with anthracyline therapy [7,8].

Serial multiple gated acquisition scans (MUGA) are widely used to monitor cardiac dysfunction in breast cancer patients. However, with the improvement in both spatial and temporal resolution of cardiac magnetic resonance (CMR) over the past decade, it has now become the gold standard for the non-invasive assessment of left ventricular (LV) systolic dysfunction. Additionally, late gadolinium enhancement (LGE) can detect myocardial scarring. Although frequently used in the assessment of dilated cardiomyopathies secondary to ischemia or myocarditis [9], little is known about the utility of CMR in the assessment of trastuzumab induced cardiomyopathy. We report a case series of trastuzumab induced myocarditis characterized by left ventricular dysfunction and focal epicardial LGE using CMR imaging.

## Methodology

### Patient population

Between 2005–2006 inclusive, 160 breast cancer patients who received trastuzamab in addition to anthracyline based adjuvant therapy were identified at a tertiary care oncology centre. All patients received FEC (5-fluorouracil, epirubicin and cyclophoshamide) for a total of 6 cycles. The mean duration between completion of chemotherapy and initiation of trastuzumab was 2 ± 1 months. Of the total population, 10 patients were identified with trastuzumab induced cardiomyopathy based on LV ejection fraction less than 40% on either serial MUGA or echocardiography. The medical records of all 10 patients were extensively reviewed for baseline demographic data. The retrospective study was approved by the local institutional review board.

### Cardiac MRI

CMR was performed on all 10 patients using a 1.5 T scanner (Avanto, Siemens, Erlangen, Germany). Morphologic images in the cardiac short axis, 4 chamber long axis and 2 chamber long axis planes were acquired using IR-prepared dark blood HASTE sequences (TR 600 ms, TE 26 ms, 6 mm slice thickness, 1.8 mm interslice gap). In the same planes, cine-CMR was performed using a breath-hold balanced steady state free precession sequence (TrueFISP, TR 42 ms, TE 1.2 ms, FA 70°, 6 mm slice thickness, matrix 192 × 174). The cine-CMR short-axis images encompassed the entire LV from the base to the apex (stack of 10 sequential short-axis slices; TR 64 ms, TE 1 ms, FA 80°, 8 mm slice thickness, 1.6 mm interslice gap, matrix 192 × 132) to obtain a left ventricular ejection fraction (LVEF). Late gadolinium enhancement was performed after 10 minutes of 0.2 mmol/kg injection of Gadolinium (Gd-DTPA, Magnevist, Schering, Germany) using a T1-weighted IR-prepared multislice TrueFISP sequence with magnitude and phase sensitive reconstruction. Images were acquired sequentially in the short axis, followed by horizontal and vertical long axis images (TR 700 ms, TE 1.0 ms, FA 40°, 8 mm slice thickness, 1.6 mm interslice gap, matrix 192 × 144).

## Results

The total population included 10 patients (mean age 40 ± 8 years, range 27 to 56 years) with normal LVEF at baseline and preserved systolic function following administration of anthracycline based chemotherapy using serial MUGA scans (Table 1). Cardiovascular risk factors, dose and frequency of chemotherapy, concomitant use of radiation therapy, and duration of trastuzumab therapy (3–5 months) were similar in the entire patient cohort (Table 1).

**Table 1:** Clinical Characteristics of Patient Population (n = 10)

***Case No*.**	***Age***	***CV risk***	***Radiotherapy***	***Baseline LVEF (%)***	***Post Chemothx LVEF (%)***	***Trastuzumab Duration***
1	33	None	Yes	55	52	4 months
2	41	HTN	Yes	60	58	5 months
3	27	None	Yes	54	56	4 months
4	39	None	Yes	65	62	3 months
5	44	Lipids	Yes	58	55	4 months
6	38	None	Yes	56	52	5 months
7	56	HTN	Yes	60	62	3 months
8	45	HTN	Yes	54	55	4 months
9	32	None	Yes	55	52	5 months
10	40	HTN	Yes	60	58	4 months

CV, cardiovascular; LVEF, left ventricular ejection fraction; chemothx, chemotherapy; HTN, hypertension.

**Figure 1:**
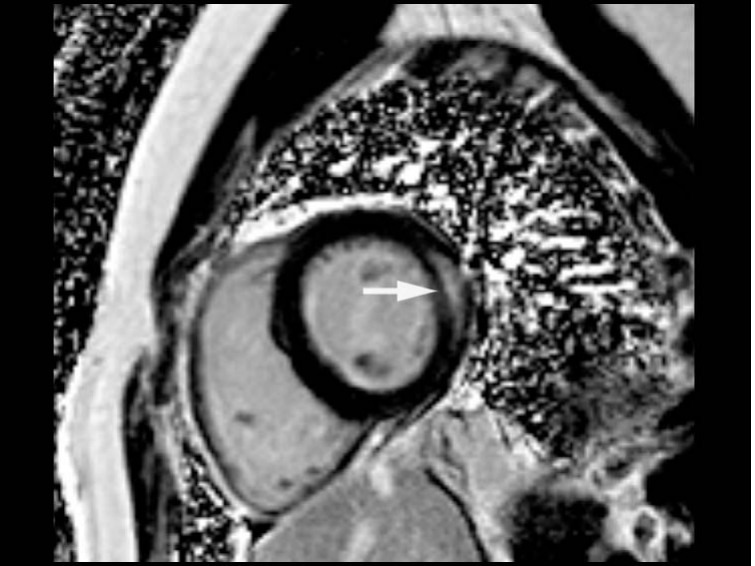
Short axis phase sensitive reconstructed IR-TrueFISP image through the mid-ventricle demonstrates subepicardial linear late gadolinium enhancement (arrow) in the lateral wall of a patient who had received Trastuzumab.

At the time of diagnosis of trastuzumab induced cardiomyopathy, the left ventricular cavities were dilated with moderate to severe global LV systolic dysfunction on CMR (Table 2). The mean LVEF was for the total population was 29 ± 4% (Table 2). Subepicardial linear LGE was present in the lateral portion of the left ventricles in all 10 patients suggesting the presence of trastuzumab induced myocarditis (Figure 1).

**Table 2:** CMR findings of patient population (n = 10)

***Case No*.**	***Age***	***LVEF (%)***	***Delayed enhancement***	***Medical treatment***	***6 month f/u LVEF (%)***
1	33	32	Lateral, septal	ACEI, beta blockers	55
2	41	28	Lateral	ACEI, beta blockers	60
3	27	30	Lateral	ACEI, beta blockers	40
4	39	25	Lateral	ACEI, beta blockers	65
5	44	35	Lateral, septal	ACEI, beta blockers	60
6	38	30	Lateral	ACEI, beta blockers	40
7	56	25	Lateral	ACEI, beta blockers	30
8	45	24	Lateral	ACEI, beta blockers	55
9	32	30	Lateral	ACEI, beta blockers	35
10	40	34	Lateral	ACEI, beta blockers	58

LVEF, left ventricular ejection fraction; ACEI, angiotensin converting enzyme inhibitor.

Following the discontinuation of trastuzumab, 6 patients have recovered normal LV systolic function, while 4 patients have persistent LV dysfunction at six month followup, despite appropriate heart failure medications including ACE inhibition and beta blockade (Table 2). The linear LGE-CMR findings persisted at 6 months in all patients despite improvement in LVEF in over half of the patients.

## Discussion

An increasing understanding of the biology of breast cancer has lead to the identification of novel therapeutic targets. The HER2 receptor is a member of the epidermal growth factor receptor family of transmembrane tyrosine kinases and is normally involved in the regulation of cell proliferation. Increased expression of HER2 is detected in 25–30% of breast cancers and is associated with poorly differentiated tumors with a high proliferative rate, positive axially lymph nodes and decreased expression of estrogen and progesterone receptors [3]. These characteristics are associated with an increased risk of disease recurrence and death due to breast cancer [3].

Trastuzumab (Herceptin) is a relatively new chimerized monoclonal antibody that targets the extracellular portion of the HER2 membrane protein. Previous studies have demonstrated that trastuzumab provides considerable therapeutic benefits, when added to conventional anthracycline chemotherapy, in decreasing the rates of disease recurrence and death in HER2 overexpressed metastatic breast cancers [4-6]. Despite its therapeutic benefit, trastuzumab is cardiotoxic with a 5% prevalence of cardiomyopathy when used as monotherapy and 10–15% prevalence of cardiomyopathy when used in combination with anthracyclines [7,8].

Various theories have been suggested regarding the possible pathogenetic origin of trastuzumab induced cardiomyopathy. These include potentiation of anthracycline induced cardiotoxicity and immune mediated destruction of cardiomyocytes [10]. There is increasing evidence as well supporting a direct toxic effect of HER2 blockade on the myocardial tissue. HER2 signaling appears to play an important role in the embryonic cardiac development and cardioprotection; the blockade of this pathway by trastuzumab can also lead to myocardial inflammation and damage [11].

The use of CMR for the non-invasive characterization of trastuzumab induced myocarditis is novel. Whereas CMR has become the standard diagnostic test in the evaluation of suspected myocarditis [9,12], to date, there are no reports in the literature describing the utility of LGE for the diagnosis of trastuzumab induced cardiomyopathy. The current report demonstrated the common finding of LGE of the subepicardial lateral wall in all patients, which may be a typical distribution and location of myocarditis in this drug induced cardiomyopathy. Although 6 patients recovered LVEF by CMR with appropriate therapy for CHF, it is of interest that the LGE findings persisted up to 6 months in all patients, suggesting persistent injury to the myocardium. A limitation of the current retrospective study however is the small number of individuals. A larger prospective series may enable us to make more substantive conclusions regarding the role of LGE-CMR in the diagnosis and prognosis of this patient population.

## Conclusion

Late gadolinium enhancement using CMR is a novel way of detecting early changes in the myocardium due to trastuzumab induced cardiotoxicity. Future studies are required to validate identification of positive delayed enhancement using CMR as a subclinical marker for future LV dysfunction in this select population. Early detection of LV dysfunction using LGE-CMR may allow one to adjust treatment with trastuzumab prior to the development of irreversible heart failure.

## Competing interests

The author(s) declare that they have no competing interests.
